# Inhibition of endoplasmic reticulum stress improves coronary artery function in the spontaneously hypertensive rats

**DOI:** 10.1038/srep31925

**Published:** 2016-08-23

**Authors:** Soo-Kyoung Choi, Mihwa Lim, Seon-Hee Byeon, Young-Ho Lee

**Affiliations:** 1 Department of Physiology, College of Medicine, Brain Korea 21 Plus Project for Medical Sciences, Yonsei University, Seoul, Korea

## Abstract

Endoplasmic reticulum (ER) stress has been shown to play a critical role in the pathogenesis of cardiovascular complications. However, the role and mechanisms of ER stress in hypertension remain unclear. Thus, we hypothesized that enhanced ER stress contributes to the maintenance of hypertension in spontaneously hypertensive rats (SHRs). Sixteen-week old male SHRs and Wistar Kyoto Rats (WKYs) were used in this study. The SHRs were treated with ER stress inhibitor (Tauroursodeoxycholic acid; TUDCA, 100 mg/kg/day) for two weeks. There was a decrease in systolic blood pressure in SHR treated with TUDCA. The pressure-induced myogenic tone was significantly increased, whereas endothelium-dependent relaxation was significantly attenuated in SHR compared with WHY. Interestingly, treatment of ER stress inhibitor normalized myogenic responses and endothelium-dependent relaxation in SHR. These data were associated with an increase in expression or phosphorylation of ER stress markers (Bip, ATF6, CHOP, IRE1, XBP1, PERK, and eIF2α) in SHRs, which were reduced by TUDCA treatment. Furthermore, phosphorylation of MLC_20_ was increased in SHRs, which was reduced by the treatment of TUDCA. Therefore, our results suggest that ER stress could be a potential target for hypertension.

Hypertension is one of the leading causes for cardiovascular disease worldwide. A contributing factor to hypertension is elevated vascular tone in small arteries and arterioles. Although increasing number of studies have been investigating the augmented vascular tone in the hypertension, the exact mechanism remains unclear. Recently, few studies have reported a link between endoplasmic reticulum (ER) stress and hypertension. However, it is limited to show endothelium derived contracting factor (EDCF)-mediated signaling in aorta^1^ and carotid artery^2^.

The ER is a specialized organelle responsible for the synthesis, assembly, folding, and sorting of proteins. When ER homeostasis is perturbed, the unfolded protein response occurs to perform corrective functions that challenges ER function, such as inflammation, disruption of calcium homeostasis, and alterations in cellular redox status, leads to an accumulation of misfolded proteins^3^,^4^. To re-establish ER homeostasis, cells activate the unfolded protein response (UPR) involving attenuation of translation, up-regulation of ER chaperones, increased protein degradation, transcriptional activation^5^,^6^. The UPR is initiated by activation of three distinct sensors at the ER membrane, including inositol-requiring enzyme-1 (IRE1), PKR-like ER kinase (PERK), and activating transcription factor-6 (ATF6)^7^. Engagement of UPR sensors triggers changes in downstream signaling such as X-box binding protein 1 (XBP1), CCAAT-enhancer-binding protein homologous protein (CHOP), eukaryotic translation initiation factor 2 subunit alpha (eIF2α), which leads to the up-regulation of various UPR target genes to restore ER homeostasis^8^. Recently, alterations of the function in the ER have been reported as a contributing factor to pathophysiology of several diseases including cancer^9^, neurodegenerative diseases^10^,^11^, and diabetes^12^,^13^.

Tauroursodeoxycholic acid (TUDCA) is a hydrophilic bile acid that is normally produced endogenously in the liver^14^. TUDCA has long been used as a bile acid replacement therapy for the treatment of cholestasis and hepatocellular necrosis^15^. Recently, its effects have been reported in pulmonary hypertension^16^ and cardiovascular disease such as myocardial contractile dysfunction^17^, myocarditis^16^,^17^,^18^. However, the modulatory effects of TUDCA in hypertension remain unclear. Therefore, the present study investigated whether ER stress is increased in the coronary arteries of spontaneously hypertensive rats (SHRs) and treatment of TUDCA could alleviate the increased ER stress and normalize the elevated blood pressure in SHRs.

## Results

### Effect of ER stress Inhibition on Body Weight and Blood Pressure

**Figure 1:**
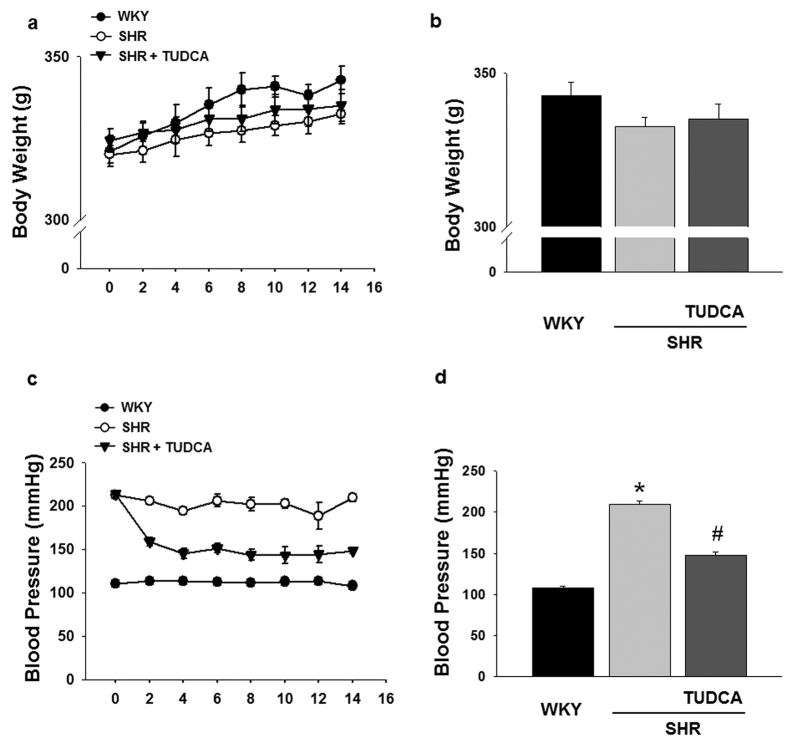
Effects of the ER stress inhibition on body weight and blood pressure in WKYs and SHRs. (**a,b**) Comparison of body weight between WKYs and SHRs with or without TUDCA (n = 5) showing time course of changes (**a**) and at the end of the experiment (**b**). (**b**) Comparison of systolic blood pressure levels between WKYs and SHRs with or without TUDCA (n = 5) showing time course of changes (**c**) and at the end of the experiment (**d**). *p < 0.05 for WKYs vs. SHRs, ^#^p < 0.05 for SHRs vs. SHRs with TUDCA.

There were no significant differences in body weight between groups (WKY: 342.75 ± 4.13 g, SHR: 332.5 ± 3.06 g, SHR + TUDCA: 335.0 ± 4.916 g; at the end of experiments, Fig. 1a,b). Blood pressure was significantly higher in SHRs compared to WKYs. Interestingly, ER stress inhibitor significantly reduced the blood pressure in SHRs (WKY: 107.75 ± 2.49 mmHg, SHR: 209.25 ± 4.46 mmHg, SHR + TUDCA: 148.0 ± 3.24 mmHg; at the end of experiments, Fig. 1c,d).

### Effect of ER Stress Inhibition on Myogenic Response and Endothelium Dependent Relaxation in Coronary Arteries

**Figure 2:**
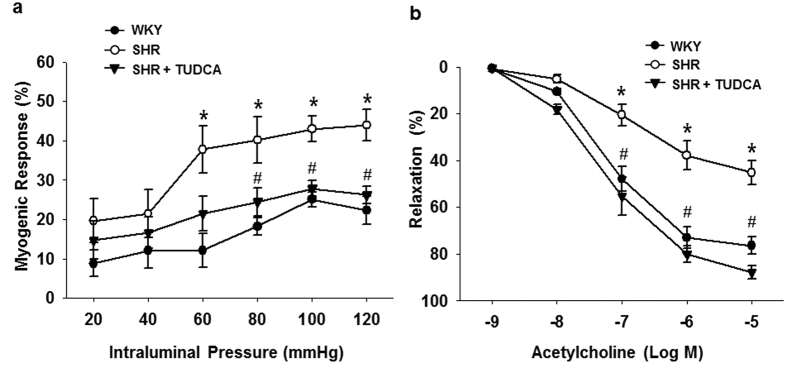
Effects of the ER stress inhibition on myogenic response and endothelium-dependent relaxation in coronary arteries from WKYs and SHRs. (**a**) The summarized data for the effects of ER stress inhibition on pressure-induced myogenic response. Changes in inner diameter were measured in response to 20 mmHg stepwise increases in intraluminal pressure in the K-H solution (active diameter) or Ca^2+^ free K-H solution (passive diameter). (n = 5). (**b**) Summarized data for endothelium-dependent relaxation in response to cumulative doses of acetylcholine (10^−9^ to 10^−5^ mol/L) in the coronary arteries pre-contracted with U-46619 (10^−7^ mol/L). *p < 0.05 for WKYs vs. SHRs, ^#^p < 0.05 for SHRs vs. SHRs with TUDCA (n = 5).

The myogenic response and endothelium-dependent relaxation were measured to evaluate the vascular reactivity in isolated coronary arteries. Myogenic response was significantly augmented in coronary arteries from SHRs compared to WKYs and was significantly reduced by the treatment of ER stress inhibitor (Fig. 2a). Endothelium-dependent relaxation was significantly impaired in coronary arteries from SHRs compared to WKYs. Interestingly, treatment of TUDCA significantly improved endothelium-dependent relaxation in coronary arteries from SHRs (Fig. 2b).

### Effect of ER stress Inhibition on Expression and Phosphorylation of ER Stress Sensors

**Figure 3:**
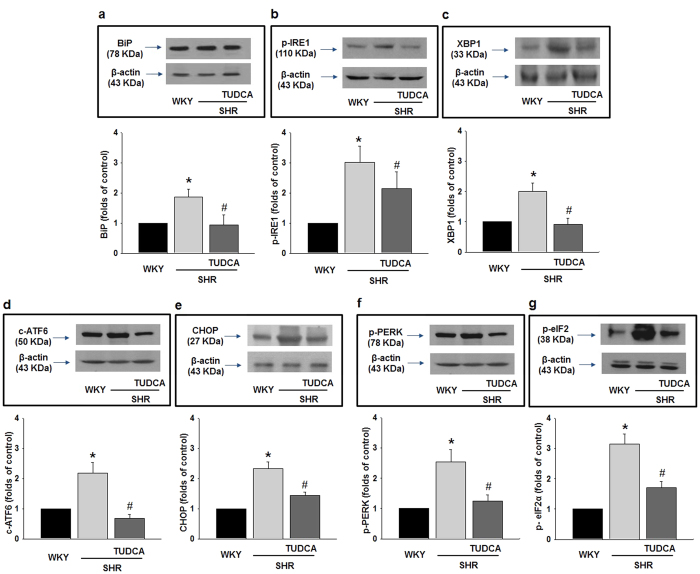
Effects of TUDCA on the expression or phosphorylation of ER stress sensors. Representative western blot analysis and quantitative data for BiP expression (**a**), phosphorylated IRE1 and XBP1 expression (**b,c**); cleaved ATF6 and CHOP (**d,e**); phosphorylated PERK and phosphorylated eIF2α (**f,g**) in all groups. (n = 4) *p < 0.05 for WKYs vs. SHRs, ^#^p < 0.05 for SHRs vs. SHRs with TUDCA.

**Figure 4:**
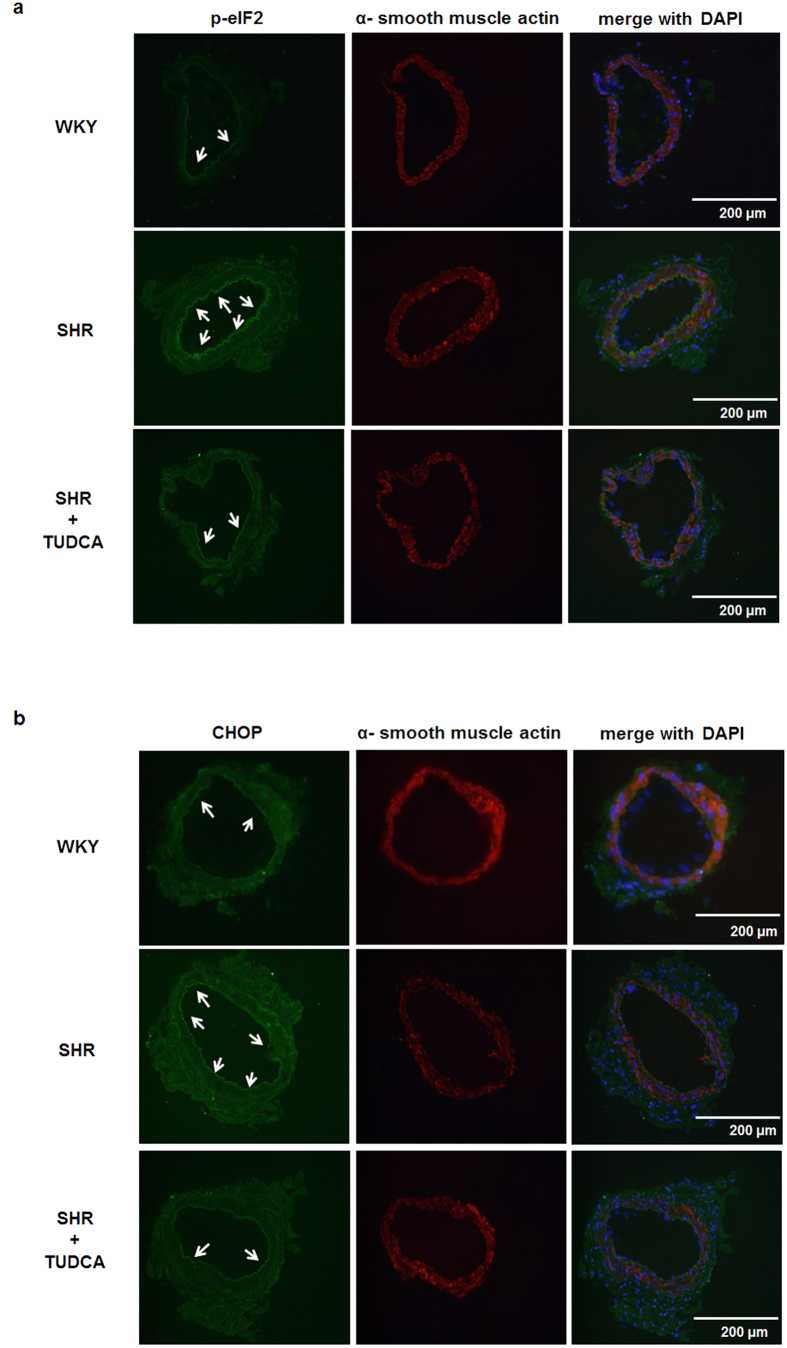
Effects of TUDCA on the expression or phosphorylation of ER stress markers in the isolated coronary arteries. Immunofluorescence of phosphorylated eIF2α and α-smooth muscle actin (**a**), and CHOP and α-smooth muscle actin (**b**). The arrows show positive staining.

To determine whether ER stress is increased in the coronary arteries of SHRs and whether the treatment of TUDCA decrease ER stress, we observed the expression and/or phosphorylation of ER stress marker proteins. We found that expression of BiP was significantly enhanced in SHRs compared to WKYs (Fig. 3a). And phosphorylation of IRE1 and expression of XBP1 were increased in SHRs (Fig. 3b,c). Interestingly, inhibition of ER stress with TUDCA decreased up-regulation of these sensors (Fig. 3a–c). Furthermore, cleaved ATF6 and expression of CHOP were increased in coronary arteries of SHR, which is normalized by treatment of TUDCA (Fig. 3d,e). Phosphorylated PERK and eIF2α were also increased in SHRs compared to WKYs and were suppressed by the treatment with TUDCA (Fig. 3f,g). These results provide evidence that treatment with TUDCA effectively inhibited ER stress in SHRs and all three branches of UPR process were associated the ER stress in SHRs. To confirm western blot data, we performed immunofluorescence staining of ER stress markers in isolated coronary arteries. We found that phosphorylation of eIF2α (Fig. 4a) and CHOP (Fig. 4b) were significantly increased in the isolated coronary arteries from the SHRs compared to arteries from WKYs. In accordance with western blot analysis, inhibition of ER stress with TUDCA decreased up-regulation of these marker proteins (Fig. 4a,b).

### Effect of ER stress Inhibition on Phosphorylation of Myosin Light Chain

**Figure 5:**
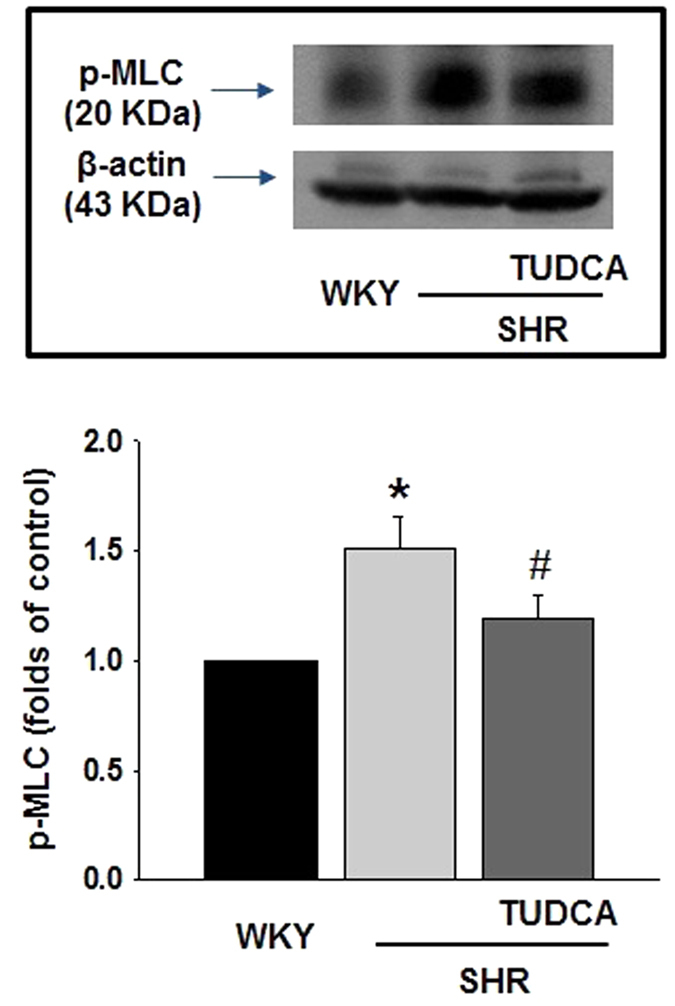
Effects of TUDCA on the phosphorylation of MLC_20_ at Ser^19^. The representative western blot analysis and quantitative data for phosphorylation of MLC_20_. (n = 4) *p < 0.05 for WKYs vs. SHRs, ^#^p < 0.05 for SHRs vs. SHRs with TUDCA.

Since vascular tone is determined by phosphorylation of MLC_20_, we investigated whether MLC_20_ phosphorylation is increased in coronary arteries from SHRs and affected by treatment of TUDCA. The phosphorylation of MLC_20_ at Ser^19^ in SHRs was significantly augmented compared to WKYs, and reduced by treatment of TUDCA (Fig. 5).

## Discussion

In the present study, we found that ER stress contributes to vascular dysfunction in hypertension. The main findings of this study are (1) ER stress inhibitor, TUDCA, decreased blood pressure in SHRs; (2) myogenic response and endothelium-dependent relaxation were impaired in coronary arteries from SHRs and the inhibition of ER stress normalized myogenic response and endothelium-dependent relaxation in SHRs; (3) the ER stress sensors were up-regulated in the coronary arteries from SHRs, which were reduced by treatment of TUDCA; (4) phosphorylation of MLC_20_ was increased in SHRs, and were decreased by TUDCA treatment. These results suggest that increased ER stress is responsible for coronary artery dysfunction in SHRs, likely through up-regulation of all three UPR branches including PERK, ATF6, and IRE1. Furthermore, these results were associated with ER stress inhibition decreased phosphorylation of MLC_20_ which is the determinant of vascular tone.

Hypertension is the major risk factor of cardiovascular disease. It has been suggested that an increased peripheral resistance is the fundamental hemodynamic disorder in hypertension^19^. The increased peripheral resistance of hypertension is due to a defective mechanism in the contractility of vessels and structural alterations of vessel walls. Several studies reported that calcification and consequent structural changes of the vessel wall occur in coronary artery disease^20^,^21^. In the present study, we focused on investigating the vascular contractility in coronary arteries of hypertensive animals. Vascular reactivity is mainly controlled by myogenic response and endothelium-dependent relaxation mechanisms. Myogenic response is defined by vasoconstriction in response to an increase of intravascular pressure and vasodilation in response to a decrease in intravascular pressure, and is an intrinsic vascular response which plays an important role in the local regulation of blood flow^22^. Previous studies have reported that arterial function is impaired due to the elevated myogenic response in hypertensive rats^23^,^24^. Interestingly, in the present study, we observed that enhanced myogenic response in coronary artery of SHRs associated with increased ER stress sensors. Thus, ER stress inhibition using pharmacological approach reduced enhanced myogenic response. Vascular reactivity is also regulated by endothelium-dependent relaxation. In this study, we found that endothelium-dependent relaxation was impaired in coronary arteries from SHRs, which is in accordance with previous reports^1^,^25^. Moreover, treatment of ER stress inhibitor, TUDCA normalized impaired endothelium-dependent relaxation in SHRs. To further demonstrate which UPR components are involved in the ER stress induced vascular dysfunction in SHRs, we observed expression and phosphorylation of the ER stress sensor proteins. The BiP is an intracellular chaperone, and regulates the UPR to protect cells from apoptosis under stress condition^26^. In the present study, we observed that BiP was up-regulated in SHR and significantly reduced by the treatment with TUDCA. It has also been established that three unique pathways comprise the UPR. In each branch, IRE1, PERK, and ATF6 sense abnormal conditions in the ER lumen and transmit the information across the membrane into the cytosol where a series of transcription factors carry information to the nucleus^26^. In the current study, we observed that enhanced phosphorylation of IRE1 and its downstream XBP1 expression in SHRs compared to WKYs. Furthermore, phosphorylation of PERK and eIF2α were also elevated in SHRs, which is reduced by the treatment with TUDCA. In addition, we observed that cleaved ATF6 and CHOP are increased in coronary arteries of SHRs, which is also decreased by the treatment of TUDCA. It has been observed the contribution of each ER stress sensor branches, ATF6-CHOP, IRE1-XBP1, and PERK-eIF2α in several diseases^27^,^28^. However, is has not been reported which ER stress sensors are related with vascular dysfunction in coronary arteries of SHR. Thus, this is the first study to demonstrate the UPR mechanisms responsible for coronary artery dysfunction in SHRs. We clarified that all the branches of UPR sensors contribute vascular dysfunction on SHRs.

Vascular smooth muscle contraction is primarily regulated by phosphorylation at Ser^19^ of the MLC_20_^29^. And it has been reported that MLC_20_ phosphorylation is increased in arteries of hypertensive animal models^30^,^31^. However it is not known that ER stress is involved in the regulation of MLC_20_ phosphorylation. In the present study, we observed that enhanced phosphorylation of MLC_20_ was significantly reduced by the treatment of TUDCA. However, direct link between MLC_20_ phosphorylation and ER stress signaling was not investigated in this study. Further experiments are needed to define how the ER stress affects phosphorylation of MLC_20_.

In the present study, we found that ER stress was associated with vascular dysfunction in coronary arteries of SHRs. The inhibition of ER stress by TUDCA reduced blood pressure in SHRs, which are associated with improvement of vascular reactivity. These data are in accordance with the previous study demonstrated that ER stress induction may contribute to increases in blood pressure, which is associated with the enhanced EDCF response in the aorta of the SHRs^1^. In addition, it has been also reported that inhibition of ER stress with Berberine improves endothelial function in carotid arteries of SHRs^2^. However, limitations of these studies are that they showed the involvement of ER stress in large vessels such as aorta^1^ and carotid artery^2^ using tension measurements. In contrast, we performed the study with coronary arteries using pressurized arteriograph which has more physiological relevance. In conclusion, we suggest that ER stress is exacerbated in SHRs and inhibition of ER stress normalizes increased blood pressure, augmented myogenic responses, and impaired endothelium-dependent relaxation, due to decreased phosphorylation of MLC_20_ which are associated with reduction of ER stress sensors such as Bip, ATF6, CHOP, IRE1, XBP1, PERK, and eIF2α pathways.

The major limitation of our study is that the improvement of vascular function in SHRs treated with TUDCA may be secondary to the systemic administration of TUDCA which acts in the central nervous system. It has been suggested that systemic administration of ER stress inhibitors could reduce blood pressure due to the inhibition of ER stress in the subfornical organ (SFO)^32^,^33^. Thus, it is not clear whether TUDCA have a primary effect on the reduction of blood pressure in SHRs. Therefore, additional experiments are needed to describe the local effects and underlying mechanisms. Furthermore, how the UPR components affect the MLC_20_ phosphorylation was not examined in the present study. Thus, further studies are needed to investigate whether ER stress inhibition affects the regulation of myosin light chain kinase (MLCK) or myosin light chain phosphatase (MLCP), which determines the level of MLC_20_ phosphorylation. Despite the limitations of this study, this is the first study to show the effect of ER stress inhibition in the coronary artery dysfunction in SHRs. Moreover, we showed which UPR pathways are related with the vascular dysfunction and consequent high blood pressure in SHRs. Thus, our study provides new approach for the treatment of hypertension.

## Methods

All experiments were performed according to the Guide for the Care and Use of Laboratory Animals published by US National Institutes of Health (NIH publication, 8^th^ Edition, 2011) were approved by the Ethics Committee and the Institutional Animal Care and Use Committee of Yonsei University, College of Medicine.

### Animal Models

Sixteen to eighteen weeks old male wistar kyoto rats (WKYs) and spontaneously hypertensive rats (SHRs) were used in this study. Rats were divided into three groups: (1) WKYs with no treatment (n = 10); (2) SHRs with no treatment (n = 10); (3) SHRs who received ER stress inhibitor (n = 10, TUDCA, 150 mg/kg/day, intra-peritoneal injection for two weeks). The body weight and blood pressure were measured every other day. At the end of the treatment period rats were anesthetized with isoflurane via a nose cone for euthanasia. The depth of anesthesia was monitored by pinching the toes, and no reaction was taken as a confirmation of proper anesthesia. Then, hearts were isolated and immediately placed in ice-cold Krebs-Henseleit (K-H) solution (composition in mmol/L: NaCl, 119; CaCl_2_, 2.5; NaHCO_3_, 25; MgSO_4_, 1.2; KH_2_PO_4_, 1.2; KCl, 4.6; and glucose, 11.1) and processed for the further studies.

### Blood Pressure Measurement

Blood pressure (BP) was measured in the warmed, conscious, restrained state by using the tail-cuff method using a photoplethysmography blood pressure monitoring system (BP-2000, Visitech Systems, Apex, NC, USA). Animals were trained to the apparatus for 1 week, before recordings were collected. At each time point, at least 10 readings were obtained for each rat and the average of the readings taken.

### Preparation of Isolated Coronary Arteries

After 2 weeks of treatment, rats were sacrificed and heart was immediately excised and placed into ice-cold K-H solution. The left anterior descending coronary arteries were isolated, dissected (segments length ~2 mm), cannulated with glass micropipettes, and perfused with K-H solution bubbled with a 95% O_2_ + 5% CO_2_ gas mixture to maintain pH of 7.4 at 36 °C. The vessels were pressurized to 40 mm Hg using pressure-servo control perfusion systems (Living Systems Instruments, St Albans, VT, USA) for a 40-minute equilibration period. A video image analyzer, as described previously^34^, monitored the vessel diameter. Intraluminal pressure was increased from 20 to 120 mmHg in a stepwise manner to measure myogenic responses. At the end of the experiments, vessels were incubated with a calcium-free K-H solution containing calcium chelating agent, ethylene glycol tetraacetic acid (EGTA, 1 mM) and Ca^2+^ channel blocker, nifedipine (5 μM) to determine passive diameter. Myogenic response is calculated as the percentage between active and passive diameters. To determine the endothelium-dependent relaxation, pressurized arteries were pre-contracted with thromboxane agonist (U-46619, 10^−7^ mol/L), and then cumulative concentrations (10^−9^ to 10^−5^ mol/L) of acetylcholine were applied.

### Western Blot Analysis

Freshly isolated coronary arteries from all of the groups were immediately frozen in liquid nitrogen and then homogenized in ice-cold lysis buffer, as previously described^35^. Western blot analysis was performed for BiP (binding immunoglobulin protein; 1:1000 dilution; Cell signaling, Danvers, MA, USA), phosphorylated IRE1, XBP-1 (1:1000 dilution; Cell signaling, Danvers, MA, USA), phosphorylated-eIF2α (1:1000 dilution; Abcam, Cambridge, MA, USA), cleaved ATF6 (1:1000 dilution; Cell signaling, Danvers, MA USA), CHOP (1:1000 dilution; Cell signaling, Danvers, MA, USA), and phosphorylated PERK (1:1000; Cell Signaling, Danvers, MA, USA), and phosphorylated 20 KDa myosin light chain (MLC_20_, 1:1000; Cell Signaling, Danvers, MA, USA). Blots were stripped and then reprobed with the β-actin antibody (1:2000 dilution; Santa Cruz Biotechnology, Dallas, TX, USA) to verify the equal loading between the samples.

### Immunofluorescence staining of ER stress markers in coronary artery

The expression of CHOP and phosphorylated eIF2α in the isolated coronary arteries were measured using immunofluorescence staining as described previously^30^. Briefly, after 2 weeks of treatment, rats were sacrificed and coronary arteries were isolated, as described above, were immediately embedded in OCT, and were frozen in liquid nitrogen. Serial 5-μm-thick sections, were collected. For immunofluorescence staining, the sections were incubated with α- smooth muscle actin antibody and CHOP or phospho-eIF2α at 4 °C overnight. Antibody binding was visualized by streptavidin conjugated-Alexa Fluor 555 (red) and Alexa Fluor 488 (green) (Molecular Probes, Eugene, OR, USA). Nuclei were counterstained with 4,6-diamidino-2-phenylindole (DAPI; Vectashield mounting media,Vector Laboratories, Burlingame, CA, USA).

### Drugs

The following drugs were used: U-46619 (Tocris Bioscience, Bristol, UK); acetylcholine chloride (Sigma-Aldrich, St Louis, MO, USA), TUDCA (Calbiochem, Darmstadt, Germany); the general laboratory reagents (Sigma-Aldrich, St Louis, MO, USA)

### Statistical Analysis

Results are expressed as mean ± SEM. One-way or 2-way ANOVA was used to compare each parameter when appropriate. Comparisons between groups were performed with *t*-tests when there was a significant F-statistic on a one-way or 2-way ANOVA. Values of *P* < 0.05 were considered significant. Differences between specified groups were analyzed using the Student *t* test (2-tailed) for comparing 2 groups, with *P* < 0.05 considered statistically significant.

## Additional Information

**How to cite this article**: Choi, S.-K. *et al*. Inhibition of endoplasmic reticulum stress improves coronary artery function in the spontaneously hypertensive rats. *Sci. Rep*. **6**, 31925; doi: 10.1038/srep31925 (2016).
